# Adaptive Optical Scanning Holography

**DOI:** 10.1038/srep21636

**Published:** 2016-02-26

**Authors:** P. W. M. Tsang, Ting-Chung Poon, J.-P. Liu

**Affiliations:** 1 Department of Electronic Engineering, City University of Hong Kong, 83 Tat Chee Avenue, Kowloon, Hong Kong; 2 Bradley Department of Electrical and Computer Engineering, Virginia Tech, Blacksburg, Virginia 24061 USA; 3 Department of Photonics, Feng Chia University, No. 100 Wenhwa Rd., Taichung 407, Taiwan

## Abstract

Optical Scanning Holography (OSH) is a powerful technique that employs a single-pixel sensor and a row-by-row scanning mechanism to capture the hologram of a wide-view, three-dimensional object. However, the time required to acquire a hologram with OSH is rather lengthy. In this paper, we propose an enhanced framework, which is referred to as Adaptive OSH (AOSH), to shorten the holographic recording process. We have demonstrated that the AOSH method is capable of decreasing the acquisition time by up to an order of magnitude, while preserving the content of the hologram favorably.

Capturing the holistic information of a three-dimensional scene has been a long sought technology for over a few decades. Successful attempt has been made by Enloe *et al*.^1^ who employed an analogue vidicon to capture the hologram of an object. The recorded hologram, for example, can be processed digitally to reconstruct the object image^2^. Since then numerous research works, such as phase-shifting holography (PSH)^3^,^4^,^5^, have been developed on top of this foundation using charge-coupled devices (CCDs). Despite the success of these methods, the size of the holograms, as well as the coverage of the object scene, are strictly limited by the size and resolution of the CCD camera. An effective solution to these problems have been envisioned by Poon and Korpel in the 70’s^6^ with a method now known as Optical Scanning Holography (OSH)^7^, which is capable of capturing holograms of wide-view object scenes. In addition, OSH was the first holographic technique of capturing holograms of fluorescent samples^8^. Recently there are many advancement on the technology, such as speckle reduction^9^ and resolution enhancement^10^. In passing, we want to acknowledge that recently there have been two other techniques called Fresnel incoherent correlation holography (FINCH)^11^ and Homodyne Scanning Holography^12^, a variant of optical scanning holography (OSH), that are also capable of capturing holographic information of fluorescent samples.

As the OSH system has been detailed in a lot of literatures (e.g.^7^), only a brief summary will be provided. Referring to Fig. 1 on the architecture of an OSH system, a laser beam of frequency 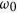 is upshifted by frequencies 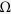 and 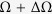 into 2 beams with acoustic-optic modulators AOM1 and AOM2, respectively. The beams emerging from the AOMs are then collimated by collimators BE1 and BE2. The outgoing beam from BE2 therefore is considered a plane wave at frequency 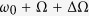 projecting onto the object though the x-y scanner, while lens L1 provides a spherical wave at 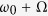 projecting on the object, 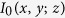. The interference between the plane wave and the spherical wave is known as the time-dependent Fresnel zone plate (TD-FZP)^7^ in OSH. It is time-dependent because there is a temporal frequency difference 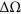 between the spherical wave and the plane wave on the object. In fact the TD-FZP oscillates at 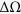. Now, the x-y scanner is used to scan the 3D object uniformly in a row by row manner. As such, each row of scanning of the object will result in a line of the hologram at the same vertical position. Along each row of scanning, photodetectors *PD*_1_ and *PD*_2_ are employed to capture the optical signal scattered by the object and the information of the heterodyne frequency 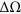 as a reference signal, respectively, and convert them into electrical signals for the lock-in amplifier. The in-phase and the quadrature (Q)-phase outputs of the lock-in amplifier give a sine hologram, 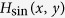, and cosine hologram, 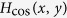, after a complete 2-D scan of the object as follows^7^:


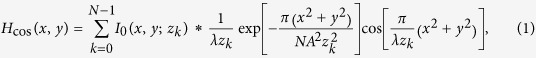


and


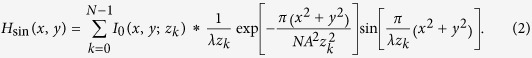


where we have assumed the 3-D object is partitioned into *N* uniformly separated image planes that are parallel to the hologram, and the *k*^th^ image plane that is located at an axial distance 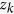 to the hologram is denoted by 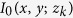. λ is the wavelength of light in free space. In Eqs (1) and (2), we have characterized the Fresnel zone plate by a numerical aperture, *NA*, which is the sine of the half-cone angle sustained by focusing lens L1. The NA determines the lateral resolution 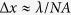 of the OSH system^7^. We have also assumed Gaussian apodization of the Fresnel zone plate by assuming that the laser beam has a Gaussian profile. Finally in Eqs (1) and (2), ∗ denotes 2-D convolution involving *x* and *y*.

From Eqs (1) and (2), we can construct a complex hologram:


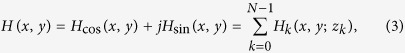


where 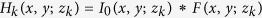, and


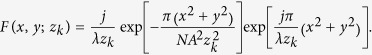


Being different from the camera-based approach, the field of vision of OSH is controlled by the angular span of the scanning system instead of being restricted by a camera of fixed sensing area. On this basis, the OSH system can be applied to capture hologram of both microscopic and wide-view scene. However, the strength of OSH also leads to its major shortcoming of requiring long acquisition time. Suppose the number of samples along the horizontal and the vertical directions are *X* and *Y* units, respectively, and *t* seconds is required to scan one hologram pixel, the time taken to capture a hologram is given by *T*_*s*_ = *XYt* seconds. For example if *X* = *Y* = 512 and *t* = 0.1 *ms*, then *T*_*s*_ = 512 × 512 × 0.1 *ms* ≈ 26 *s* which is a rather time-consuming process. A straightforward way to lower the hologram capturing time in OSH is to increase the spacing between the scan lines along the vertical direction^13^. This is equivalent to widening the gap between scan lines, leading to uniform vertical down-sampling of the hologram and distortion due to aliasing error if the sampling interval is not selected properly. To address this problem, an attempt has been made in^14^ to predict the location of each scan line according to the horizontal spatial frequency of the previous scanned line. However, the main emphasis of the method is on data compression, and less than 2 times of reduction in the scan time is achieved if the fidelity of the hologram is to be preserved favorably. There are also methods that are based on the principles of Compressive Sensing^15^, whereby a sparse representation of the hologram is obtained, for example with spiral scanning^16^. Subsequently, the hologram is reconstructed through an optimization process that is realized with multiple rounds of iterations.

In this paper we report a method, which we refer to as “Adaptive Optical Scanning Holography” (AOSH) for lowering the hologram acquisition time of classical OSH. Briefly, instead of acquiring every hologram lines through scanning the entire object scene, a prediction mechanism is incorporated to select the position of the next row to be scanned. Experimental results reveal that our proposed method is capable of reducing the number of scan rows by 5–10 times, which lowers the hologram acquisition time by a similar factor with only minor degradation on the reconstructed image. Our proposed method, together with the experimental evaluation, will be presented in the following sub-sections.

## Proposed Adaptive Optical Scanning Holography (AOSH)

As explained in the previous section, OSH acquires a hologram by scanning the object scene in a row by row manner. Each row of scanning will result in a line of hologram pixels. As a result, the hologram acquisition could be lengthy if the number of rows in the hologram is large. The objective of the ASOH method is to skip the scanning of some of the rows after the current scan line by predicting, in an on the fly manner, the vertical position of the next row to be scanned.

**Figure 2:**
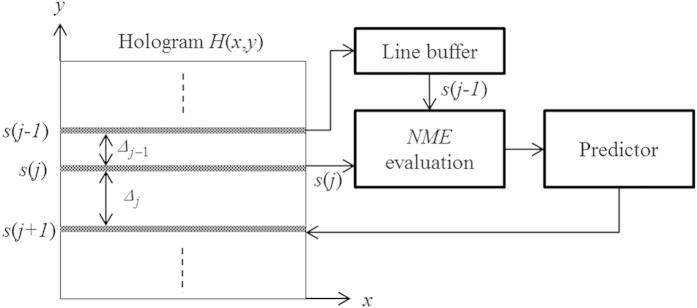
Concept of the AOSH scanning mechanism.

The concept is explained as follows. We denote the rows that will be scanned (which is unknown for the time being) with the sequence 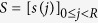 where 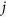 is the index of each member in 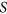, 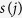 is the position of the j^th^ scan row, and *R* is the total of scanned lines. A line of the hologram 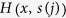 is captured for each scan row that is located at 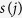. We refer to Fig. 2, showing 2 hologram lines that have been acquired previously at locations 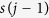 and 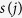. The separations between the current line 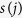 and the previous line, and the next line to be scanned are denoted by 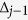 and 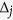, respectively. To determine the position of the next scan row 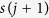, the previous line 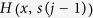 at 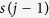 is stored in a line buffer. The previous hologram line in the line buffer and the current hologram line 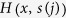 are evaluated through the Normalized-Mean-Error (*NME*) process to reflect the smoothness of the fringe patterns along the vertical direction. Next, the predictor estimate 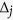, and subsequently the position 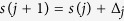 of the next scan row, assigning a wider gap between 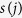 and 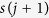 if the difference between the pair of hologram lines is small, and vice versa. The above steps are repeatedly conducted until the last row of the object scene has been scanned.

**Figure 3:**
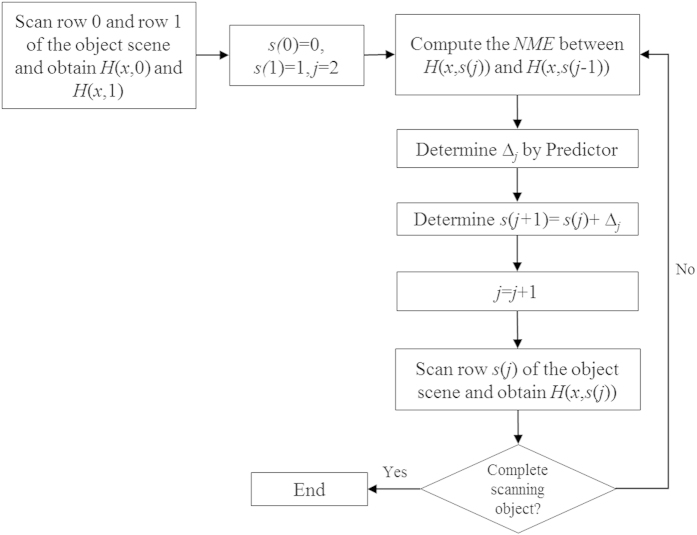
Proposed AOSH for automatic adjustment of the spacing between adjacent scan lines.

The detailed operation is shown with the flowchart in Fig. 3. Let 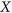 and 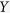 denote the number of columns and rows of the hologram, respectively. Without loss of generality, the dynamic range of the hologram pixels is assumed to be bounded within the range 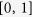.

Initially, the separation between adjacent scan rows, denoted by 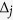, is set to ‘1’, and the first 2 rows of the object scene, i.e., 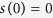 and 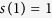 are always scanned, capturing the first two hologram lines, 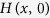 and 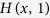. Next, the difference between the previous scanned pairs of hologram lines (i.e., 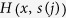 and 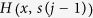) are determined with the Normalized-Mean-Error (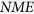) between them as


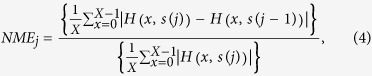


where 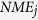 denotes the Normalized-Mean-Error between the (*j* − 1)^*th*^ and the *j*^*th*^ line. The 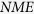, which is bounded within the range 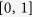, computes the average difference between correspondence pixels between 2 consecutive rows of hologram pixels. If the 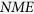 is small, it implies that the average gradient of change between the pixel values between these two lines are generally small, and vice versa. On this basis, 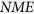 can be taken to reflect the local smoothness of the hologram along the vertical direction. We have further postulated that the change of the average gradient between consecutive lines is smooth, an assumption that is generally true in practice. As such, a small 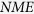 reflects that the current pair of hologram lines is similar (i.e. of a small average gradient), and the overall variation along the vertical direction should be smooth, and vice versa. Based on the general principle of Information Theory, the sampling interval should be inversely proportional to the smoothness of the signal. Along this line of reasoning, the separation between the current and the next scan row is updated by the Predictor as


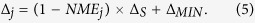


From Eq. (5), it can be seen that the minimum and maximum spacing between adjacent pairs of hologram scan lines are bounded to 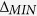 and 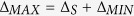, respectively. Note that 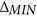 and 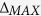 are constant parameters, and the separation between adjacent scan rows will be determined in an adaptive manner based on Eq. (5). The term 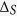 is multiplied with 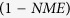, resulting in larger spacing for smooth varying hologram lines (which exhibits smaller *NME*) along the vertical direction, and vice versa. After 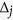 is determined, the position of the next scan row is set to


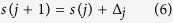


**Figure 4:**
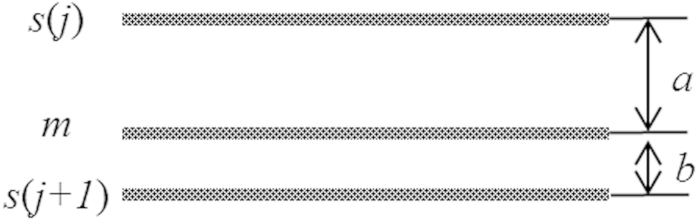
Filling a row of pixels between a pair of hologram lines.

After capturing all the hologram lines, the regions between adjacent hologram lines are filled with bi-linear interpolation as shown in Fig. 4. Given 2 hologram lines 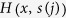 and 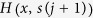, the missing line at vertical position ‘*m*’ (where 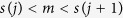) in between them is determined as


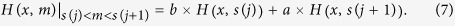


Eq. (7) implies that the value of each pixel on the missing line is obtained by the weighted sum of the hologram pixels at the pair of lines above and beneath it, with the weighting factor inversely proportional to the distance between the missing and the contributing pixels.

The factors 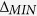 and 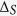 provides a tradeoff between the number of rows to be scanned (which also determines the scanning speed proportionally), and the quality of the hologram. If these 2 factors are large, fewer hologram lines will be scanned, resulting in faster capturing time and more degradation on the hologram. On the other hand if 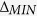 and 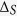 are small, a higher quality hologram will be recorded at the expenses of longer acquisition time.

## Experimental Results

**Figure 5:**
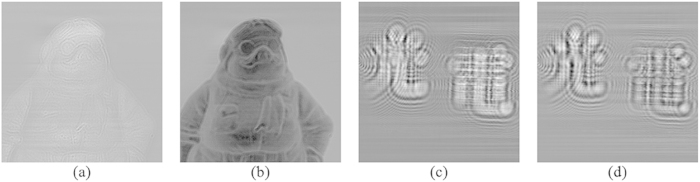
(**a**) Cosine hologram of “A”, (**b**) Sine hologram of “A”, (**c**) Cosine hologram of “B”, (**b**) Sine hologram of “B”.

**Figure 6:**
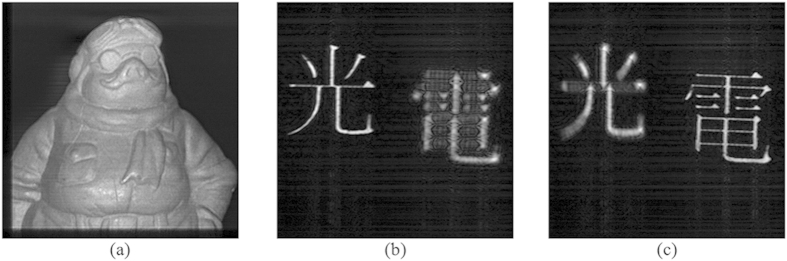
(**a**–**c**) Reconstructed images of OSH representing object “A”, “Light” of object “B”, and “Electricity” of object “B” at the corresponding focused plane, respectively.

**Figure 7:**
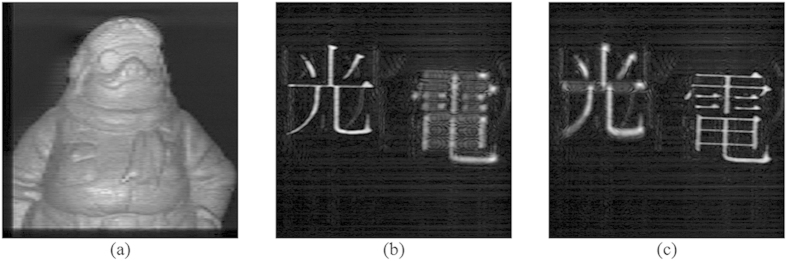
(**a**–**c**) Reconstructed images of AOSHs representing object “A”, “Light” of object “B”, and “Electricity” of object “B” at the corresponding focused plane, respectively. Apart from some blurriness, the reconstructed images of the holograms obtained by AOSH are similar to those obtained by standard OSH shown in Fig. 5(a–c).

To evaluate the AOSH method, we have applied it to capture the hologram of 2 objects “A” and “B”. Object “A” is a thin ornament located at around 20 mm from the hologram, and object “B” is comprising of a pair of Chinese characters: “Light” on the left and “Electricity” on the right that are located at 21.5 mm and 24.5 mm from the hologram, respectively. The classical OSH technique is employed to capture the holograms of the pair of objects based on the optical settings shown in Table 1. The cosine and the sine holograms of the 2 objects, and their reconstructed images at the focused plane, are shown in Figs 5(a–d) and 6(a–c), respectively. Next, we applied our proposed AOSH method to capture the hologram of object “A”, based on 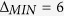 and 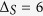, so that the maximum and minimum separation between scan lines are within the range 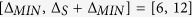. The number of lines that have been scanned to obtain the hologram for “A” and “B” are 70 and 81, respectively, implying reduction of the acquisition time by 700/70 = 10 times and 512/81 = 6.32 times as compared with the direct application of OSH, where there are 700 and 512 vertical lines for the hologram of “A” and “B”, respectively. The reconstructed images of the 2 holograms acquired by AOSH at the focused planes are shown in Fig. 7(a–c). It can be seen that apart from some blurriness, the reconstructed image are very similar to the ones obtained with the classical OSH in Fig. 6(a–c).

Apart from visual inspection, we would also like to evaluate the degradation of the reconstructed images obtained with the AOSH method in a quantitative manner. To achieve this, the fidelity of the reconstructed images, measured in Peak-Signal-to-Noise-Ratio (PSNR) as compared with the reconstructed image of the holograms acquired with classical OSH, are listed in Table 2. The PSNR is a metric that is commonly employed in evaluating the fidelity of an image after it has been modified in certain ways. From Table 2, we have observed that high PSNR of over 31db is attained for the reconstructed images of AOSH of both samples, indicating that the degradations of the images are reasonable small. These quantitative evaluations are consistent with the favorable visual quality of the reconstructed images.

## Conclusion

A framework for acquiring holograms of real world 3-D objects, which is referred to as adaptive optical scanning holography (AOSH), is proposed in this paper. As the name has implied, AOSH is an enhancement on the classical optical scanning holography technique. The major difference between the OSH and the AOSH method is that with OSH, a hologram is obtained by scanning every rows of the object scene, each resulted in a corresponding line in the hologram. In AOSH, an intelligent prediction mechanism is incorporated to conduct scanning on the rows that are likely to carry important information. As such, the overall time required to scan the entire object scene is reduced. The improvement in the hologram acquisition process is extremely important for wide-field of vision, in which case lengthy capturing time in the original OSH method is needed. We have evaluated our proposed method by applying it to capture holograms of 3-D objects. The results show that the AOSH method is around an order of magnitude faster than OSH for the tested objects, while preserving favorable quality on the reconstructed images. Such improvement is achieved with a low complexity decision process that can be easily realized with simple hardware or software implementation, and directly incorporated in the scanning process without modifying the original OSH platform. After a digital hologram has been recorded with our proposed method of AOSH, a full hologram can be generated by interpolating the missing information between adjacent hologram lines through bilinear interpolation. As only selected rows of the object scene are being scanned, AOSH also offers moderate amount of compression on the data size of the hologram. Apart from the application in OSH, we have anticipated that our proposed method could also be adopted in other holographic or optical capturing systems that are based on line scanning mechanism.

## Additional Information

**How to cite this article**: Tsang, P.W.M. *et al*. Adaptive Optical Scanning Holography. *Sci. Rep*. **6**, 21636; doi: 10.1038/srep21636 (2016).

## Figures and Tables

**Figure 1:**
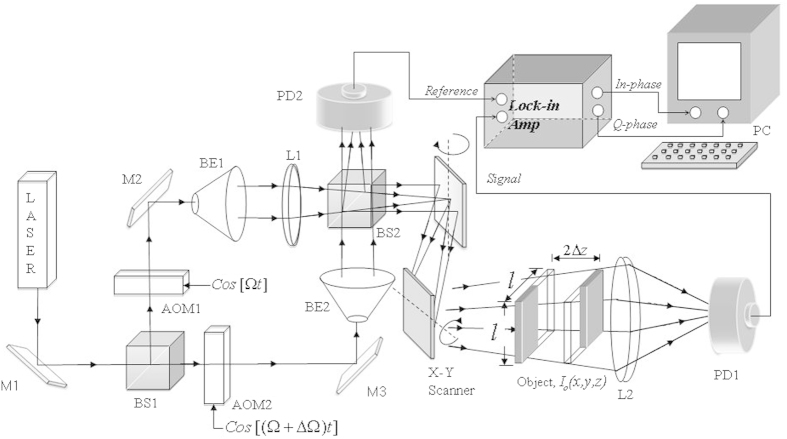
Optical scanning holography set up to record the hologram of object *I*_0_(*x, y*; *z*) (*M*’s, mirrors; *AOM1, 2*, acousto-optic modulator; *BS1,2*, beam splitter*; BE1,2*, beam expander; *L1*, focusing lens; *L2*, lens for collecting scattered light from the object into photodetector PD1; PD2, photodetector to provide heterodyne frequency Ω as a reference signal to the lock-in amplifier; PC, personal computer).

**Table 1:** Optical setting in the OSH/AOSH acquisition process.

Hologram pixel size	30 *μm* × 30 *μm*
Hologram size	700 × 700 for object “A” and 512 × 512 for object “B”.
Wavelength of optical beam	633 *nm*

**Table 2:** Fidelity of the reconstructed images (in PSNR) corresponding to the holograms obtained by Adaptive Optical Scanning Holography of the 2 samples.

Reconstructed image	Fidelity in PSNR (db)
AOSH “A” [Fig. 7a]	31.70
AOSH “B”: focused on the character “Light” [Fig. 7b]	32.47
AOSH “B”: focused on the character “Electricity” [Fig. 7c]	32.51
